# African Swine Fever Virus: A Review

**DOI:** 10.3390/v9050103

**Published:** 2017-05-10

**Authors:** Inmaculada Galindo, Covadonga Alonso

**Affiliations:** Dpto. de Biotecnología, Instituto Nacional de Investigación y Tecnología Agraria y Alimentaria (INIA), Ctra. de la Coruña km 7.5, 28040 Madrid, Spain; galindo@inia.es

**Keywords:** African swine fever virus, ASFV, virus entry, endocytosis, endosomal pathway, host cell targets, cellular responses, ER stress, apoptosis, autophagy, A179L

## Abstract

African swine fever (ASF) is a highly contagious viral disease of swine which causes high mortality, approaching 100%, in domestic pigs. ASF is caused by a large, double stranded DNA virus, ASF virus (ASFV), which replicates predominantly in the cytoplasm of macrophages and is the only member of the *Asfarviridae* family, genus *Asfivirus*. The natural hosts of this virus include wild suids and arthropod vectors of the *Ornithodoros* genus. The infection of ASFV in its reservoir hosts is usually asymptomatic and develops a persistent infection. In contrast, infection of domestic pigs leads to a lethal hemorrhagic fever for which there is no effective vaccine. Identification of ASFV genes involved in virulence and the characterization of mechanisms used by the virus to evade the immune response of the host are recognized as critical steps in the development of a vaccine. Moreover, the interplay of the viral products with host pathways, which are relevant for virus replication, provides the basic information needed for the identification of potential targets for the development of intervention strategies against this disease.

## 1. Introduction

African swine fever (ASF) is a viral disease of swine that leads to a high mortality in domestic pigs while being asymptomatic in the natural suid reservoir hosts [1,2,3]. It causes important economic losses that are unavoidable in the absence of an effective vaccine and the available methods of disease control are the quarantine of the affected area and the slaughter of the infected animals [4]. ASF is caused by the ASF virus (ASFV), a double-stranded DNA virus with a complex molecular structure. It is the only member of the *Asfarviridae* family [5] and the only DNA virus transmitted by arthropods, soft ticks of the *Ornithodoros* genus [3,6]. Soft ticks (*Ornithodoros moubata*) are involved in the sylvatic transmission cycle of the virus in Africa and *O. erraticus* in Europe. The wild boar that suffers an acute disease similar to the domestic pig appears to be relevant in the transmission cycle in Europe.

The disease caused by this virus was first identified in Kenya in the 1920s [7]. Then, it was confined to Africa until it spread to Europe in the middle of the last century, and later to South America and the Caribbean. The disease was eradicated from Europe (except of Sardinia) at the 1990s via drastic control and eradication programs. However, in 2007, the disease spread again out of Africa into the Caucasus, especially Georgia, and in 2014 it reached the eastern territory of the European Union. The latest reports of the disease include an increasing list of EU countries, Poland and the three Baltic republics [8,9] and very recently Moldova [10]. Due to the absence of vaccines with protective efficacy, ASF represents a serious threat to all European countries. The epidemiological complexity of ASF has been clearly demonstrated in eastern and southern Africa, where genetic characterization of ASFV based on sequence variation in the C-terminal region of the B646L gene encoding the major capsid protein p72, revealed the presence of 22 genotypes [11,12]. Recently, a new genotype, genotype XXIII, that shares a common ancestor with genotypes IX and X, which comprise isolates circulating in Eastern African countries and the Republic of Congo, has been described [13]. This review paper summarizes the current state of knowledge about ASFV. 

## 2. African Swine Fever Virus

ASFV is a large, enveloped virus with icosahedral morphology and an average diameter of 200 nm. The viral genome consists of a single molecule of linear, covalently close-ended, double stranded DNA. The genomes of different isolates vary in length between 170 and 190 Kbp and encode between 151 and 167 open reading frames. ASFV replication cycle is mainly cytoplasmic, but the nucleus is also a site of viral DNA synthesis at early times [14,15]. The disassembly of the lamina network close to the sites where the viral genome starts its replication and the redistribution of several nuclear proteins suggests the existence of sophisticated mechanisms to regulate the nuclear machinery during viral infection [16]. 

Transcription of viral genes is strongly regulated. Four classes of mRNAs have been identified by their distinctive accumulation kinetics—including immediate–early, early, intermediate, and late transcripts. Immediate–early and early genes are expressed before the onset of DNA replication, whereas intermediate and late genes are expressed afterwards. The presence of intermediate genes suggests a cascade model for the regulation of ASFV gene expression [17,18]. Enzymes required for DNA replication are expressed immediately after virus entry into the cytoplasm from partially uncoated core particles and using enzymes and other factors packaged in virus particles [17,18,19,20]. Virus morphogenesis takes place in the viral factories where the main late phase of DNA replication also occurs. 

## 3. Virion Structure

The ASFV particle has an icosahedral morphology composed of several concentric domains: the internal core formed by the central genome contains the nucleoid, which is coated by a thick protein layer named core shell; an inner lipid envelope surrounding the core; and finally the capsid, which is the outermost layer of the intracellular virions [21]. The extracellular virions possess an additional external envelope that is obtained when the virus buds out through the plasma membrane [22]. However, the importance of this envelope is unclear as it is not required for infectivity [23].

## 4. Viral Entry Mechanisms

The ASFV infectious cycle starts with the viral adsorption and entry into the host cell. Early studies on ASFV entry characterized this event as a low pH- and temperature-dependent process consistent with saturable and specific receptor-mediated endocytosis in Vero cells and porcine macrophages [24,25,26,27]. However, the receptor(s) for the virus still remain unknown. The limited cell tropism of ASFV suggests that a macrophage-specific receptor is required for infection. Successful infection of porcine macrophages and monocytes by ASFV correlates to the expression of the CD163 scavenger receptor, a hallmark of macrophage maturation. It was previously suggested as a potential virus receptor, as monoclonal antibodies against this molecule were able to block infection of primary alveolar macrophages [28]. However, more recent studies have demonstrated that CD163 is not necessary for infection with the Georgia 2007/1 virus isolates. Gene-edited pigs possessing a complete knockout of CD163 produced using the CRISPR/Cas9 system showed no differences in clinical signs, mortality, pathology, or viremia [29]. One conclusion from these studies was that CD163 may be necessary but insufficient for infection, suggesting that other macrophage surface proteins may participate in the infection process. 

While there is support for receptor-dependent mechanisms of viral entry, such as clathrin-mediated dynamin-dependent endocytosis [30,31], there is also evidence that ASFV exploits other mechanisms, such as phagocytosis [32] and macropinocytosis [33,34]. Also, cholesterol is required for a successful entry. These mechanisms occur both in the macrophage target cell and in Vero cells using viral isolates adapted to this cell line.

In addition, some ASFV proteins are involved in the entry mechanism such as p30, important for viral internalization, while other proteins such as p12 and p54 have been identified as potential viral attachment proteins [35,36,37].

## 5. ASFV Enters the Endosomal Pathway

**Figure 1:**
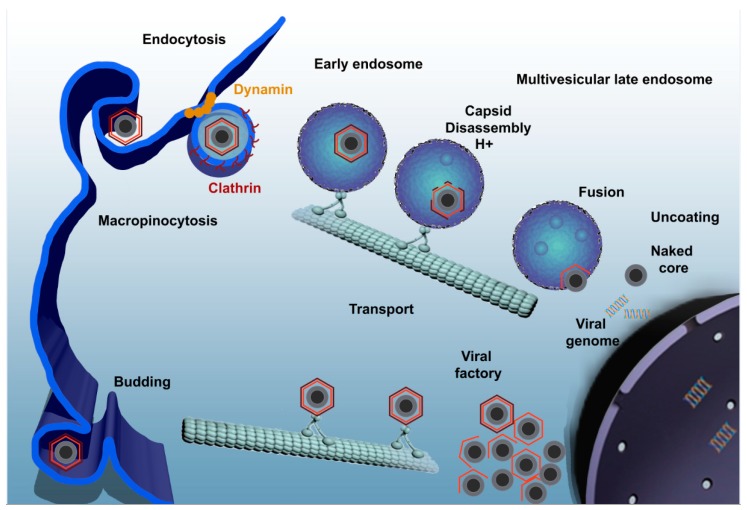
ASFV enters the host cell through a complex process involving dynamin- and clathrin-mediated endocytosis and macropinocytosis. Only few seconds later, ASFV progresses through the endocytic pathway and reaches mature endosomal compartments where viral decapsidation and fusion of the inner viral envelope with the endosomal membrane occurs. Newly synthesized virions are assembled in the viral factory and will exit the cell either by exocytosis budding at the plasma membrane or through the formation of apoptotic bodies.

ASFV infection by either pathway of entry should finally reach the endocytic pathway [38]. Once the virus has entered the endocytic pathway, it must pass through different endosome populations to achieve a successful infection (Figure 1). Endocytic pathway maturation is carefully orchestrated by proteins and lipids that are recruited to the endosomal membrane. Rab GTPase protein family is the major regulator of the endosomal maturation pathway, where each member of the Rab family is specifically located to a different endosomal compartment [39]. Incoming viruses are found in early endosomes (EE) labeled with Rab5 and EEA1 markers from very few minutes after adsorption. In fact, complete encapsidated virions are only found at the level of EE but not in other mature acidic compartments [38]. The inhibition of endosomal acidification with bafilomycin A1 prevents viral decapsidation and only under this condition it is possible to observe complete viruses inside multivesicular endosomes positive for CD63 and late endosomes expressing Rab7. In normal conditions, late endosomes harbor only viral cores lacking the capsid protein [38]. 

Viral decapsidation occurs at the acidic intraluminal pH in mature endosomal compartments between 30 and 45 min post infection (mpi). Mature endosomal compartments are multivesicular bodies expressing CD63 that are characterized by the presence of intraluminal vesicles and also, late endosomes expressing Rab7. Dependence on the endosomal maturation for sequential viral uncoating and penetration has been also described for other viruses [40,41]. Once decapsidated, virus particles expose the inner envelope which allows their interaction and subsequent fusion of this viral membrane with the limiting membrane of the endosomes and naked cores can be released into cytosol in order to start replication. This process is strongly dependent on the cholesterol efflux at the endosomal membrane. In fact, blocking cholesterol transport at this level causes retention of virions inside endosomes, inhibiting infection progression [42]. The inner envelope viral protein pE248R is also involved in viral fusion. This protein shares sequence similarity with some members of the poxviral entry/fusion complex [34]. 

Other inhibitors of endosome maturation such as wortmannin, a phosphatidylinositol 3 (PI3)-kinase inhibitor that blocks early endosome fusion, and nocodazole, an inhibitor that disturbs microtubule-dependent endosomal transport also prevent ASFV infection [38].

## 6. ASFV Gene Expression and DNA Replication

Incoming ASFV virions should reach their replication site in the perinuclear area close to the microtubule organizing center (MTOC) [43]. Immediate early and early genes are expressed before the onset of DNA replication. Both DNA chains are alternatives used as the coding strand. This is possible due to the action of several enzymes involved in viral transcription that are packed in the viral core. Following DNA replication, the transcription of intermediate and late genes begins. ASFV commits approximately 20% of its genome to encode genes involved in the transcription and modification of its mRNAs. This transcriptional machinery gives to ASFV a relative independence from its host and an accurate positional and temporal control of its gene expression [44]. The existence of a nuclear stage in the replication of ASFV DNA has been determined by in situ hybridization and autoradiography in thin sections of infected cells, although the precise role of the nucleus in viral replication remains unclear. Sedimentation analysis of replicating viral DNA in alkaline sucrose gradients has shown that small DNA fragments are pulse-labeled in the nucleus at early times in the replication of the viral DNA, whereas larger molecules are synthesized in the cytoplasm at later times. The replicative intermediates that are synthesized both in the nucleus and cytoplasm of ASFV-infected cells consist of head to head concatemers. It is possible that the nucleus may provide small transcripts or other factors required for priming virus replication or that an early stage of virus DNA replication should take place [15].

## 7. Formation of the Viral Factory

Microtubules are required for the transport of the virus to perinuclear area, where replication takes place. Integrity of microtubules is required for the formation of viral factories [45] and virus particles are found associated to stabilized microtubules at entry [46]. Also, nocodazole, which interferes with the polymerization of microtubule filaments, prevents the correct formation of factories [43,47,48,49]. Structural protein p54 interacts with the dynein motor protein during virus infection and could constitute a molecular mechanism for microtubule-mediated virus transport [43]. 

The viral factory, localized in the cytoplasm close to the nucleus, can be described as a single and large perinuclear area at the MTOC. On this site, viral proteins and DNA are accumulated and newly synthesized virions are assembled. A cage made of intermediate filament vimentin surrounds the viral factory likely to prevent the sensing of viral components into the cytoplasm and concentrate structural proteins at sites of assembly [50]. There are many features shared between aggresomes and VFs. However, neither HDAC6 nor Bag3 are required for factory formation, suggesting that aggresomes and viral factories are not the same structures [51].

Formation of viral replication sites depends on several cellular determinants. For example, Rho GTPase inhibitors produce an abnormal viral factory size with the accumulation of envelope precursors and immature virions [46]. This specialized site at the MTOC, contains viral DNA, most of the viral proteins, immature and mature virions, and also abundant virus-induced membranes. Viral factories contain precursors that develop into icosahedral intermediates by the assembly of the icosahedral capsid and the core shell domain. The last step of virion morphogenesis will be the encapsidation of DNA giving rise to mature virions. Finally, the newly formed virus leaves the factory and is transported to the cell surface by kinesin where it is released by budding [50]. Extracellular virus is covered by an additional external envelope that is acquired during this budding process [22].

## 8. ER Stress and Unfolded Protein Response

The virus modifies and interacts with cellular pathways in response to infection. The endoplasmic reticulum (ER) is an essential organelle for ASFV replication and maturation and a large number of viral proteins are synthesized in infected cells and accumulated in the ER during the viral life cycle. This process can trigger ER stress and the unfolded protein response (UPR) of the host cell as the induction of caspase 12 indicates [52]. Viruses have evolved various mechanisms to counteract these cellular responses that would limit or inhibit viral replication. This response is a regulatory program that upregulates a large number of genes, such as ER chaperones and ER-associated degradation (ERAD) components, which increase the folding capacity of the ER. ASFV induces the upregulation of the chaperones calnexin and calreticulin, but not ERp57, PDI [52], or BiP/Grp78 [52,53]. Moreover, ASFV induces selectively the transcription factor 6 (ATF6) signaling pathway of the UPR, but not the protein kinase-like ER kinase (PERK) or the inositol-requiring enzyme 1 (IRE1) pathways. Thus, the capacity of ASFV to regulate the UPR signaling cascade may prevent the effects that are detrimental to the infection, while maintaining those that are beneficial. Importantly, viral protein DP71L is involved in ATF4 downregulation and in CHOP inhibition [54]. DP71L, homolog of the neurovirulence factor ICP34.5 of HSV-1 and the cellular gene GADD34, binds to catalytic subunit of protein phosphatase 1 (PP1) and causes the dephosphorylation of eukaryotic translation initiation factor 2 alpha (eIF2 α), thereby preventing the inhibition of protein synthesis produced by ER stress and the UPR [55].

## 9. ASFV and Apoptosis

ER stress after ASFV infection is reflected by the activation of caspase 12, which follows similar temporal dynamics to the activation of mitochondrial caspase 9 and effector caspase 3 [52]. Apoptosis represents an important innate cellular mechanism to prevent virus infection, and many viruses have developed strategies for inhibiting or delaying this cellular response in turn [56]. Thus, ASFV A179L gene encodes an homolog of antiapoptotic Bcl-2 protein to prolong host cell survival until the replication of the viral genome is completed [57]. This viral Bcl-2 is expressed both at early and late times after infection and inhibits the action of several pro-apoptotic BH3-only proteins, known to be rapid inducers of apoptosis, such as activated Bid, BimL, BimS, BimEL, Bad, Bmf, Bik, Puma, and DP5 [58]. Another ASFV gene, A224L, encodes a member of the family of apoptosis inhibitors known as IAP proteins and is able to inhibit caspase activation and to promote cell survival [59,60]. Viral IAP not only blocks caspase-3 activation but also activates NF-κB [61]. It is interesting that the virus encodes an IκB-like molecule (A238L) that interferes with NF-κB activation [62]. A238L and A224L are expressed at different times during ASFV infection, this suggests that ASFV requires a low NF-κB activity at early times of infection to avoid immune responses but a higher activity at late times, probably to prevent apoptosis as the cellular systems are abused [61]. At very late stages of ASFV infection, infected cells undergo apoptosis [63] and show the characteristic morphological changes of programmed cell death, including typical membrane blebbing of the infected cell that led to the formation of numerous vesicles containing virus [45] and this could be an efficient system for virus spread.

## 10. ASFV and Autophagy

A179L, the viral Bcl2 homolog of African swine fever virus, not only interacts with pro-apoptotic Bcl2 family proteins to inhibit apoptosis but also inhibits autophagy by interacting with Beclin1 through its BH3 homology domain. ASFV is armed to counteract elimination by autophagy as other DNA viruses. An example of that is the HSV-1 ICP34.5 protein, which inhibits autophagy by targeting Beclin1 [64]. ASFV encodes a protein homologous to ICP34.5, which exerts other functions. ASFV DP71L inhibits the ER stress response activating PP1/protein phosphatase 1 [55]. However, in contrast to HSV-1 ICP34.5, it does not interact with Beclin1 [65]. 

In fact, ASFV infection does not induce LC3 activation or autophagosome formation in Vero cells [65]. Autophagy is a relevant cellular defense mechanism that allows the orderly degradation and recycling of cellular components. Autophagy eliminates intracellular pathogens and has a crucial role for innate and adaptive immune responses. Some DNA viruses, such as ASFV and HSV-1, have developed strategies to keep this cellular response under control to prevent the degradation of newly assembled virions. In contrast, most RNA viruses have been reported to induce autophagy in infected cells, and in several cases autophagy may enhance viral replication [66]. 

## 11. ASFV Egress

The mature particle is transported from the virus factories to the cell surface through a microtubule-mediated mechanism [48] depending on the motor protein conventional kinesin [67] and on the capsid protein pE120R [68]. Once on the cell surface, particles exit the host cell by budding at the membrane, acquiring an additional envelope [22]. Both intracellular and extracellular viruses are infectious but structurally and antigenically different [68], and this could have important implications in the host immune response against ASFV.

## 12. Potential Vaccines and Antivirals

Previous attempts to develop vaccines against ASFV have failed to induce protective immunity. Currently, there are several reports of the protection elicited by experimental vaccines based in live attenuated ASFV (LAV) containing single or double gene deletions in the genome [69] and other different approaches, including DNA vaccines [70,71]. Some of them might turn into successful vaccine candidates. Nowadays, a vaccine to be used in the field should meet a number of requirements. Any potential ASFV vaccine should include markers for differentiation between infected or vaccinated animals that allows for DIVA diagnostics. Also, vaccine production could not be possible because of the lack of a cell line supporting the replication of attenuated vaccine viruses without modifying the virus virulence. At present, wild boars were found to be major players of disease spread in both the Baltics and Poland. Hence, a live vaccine given as bait for these animals could be crucial to limit disease spread. In the current scenario, a vaccine would make possible to eradicate the disease together with infection surveillance by diagnostics. Available diagnostic methods allow both virus detection and also detection of antibodies for identification of survivors and asymptomatic carriers [72]. 

Until an effective vaccine is developed, the possibility of using antivirals remains. Antiviral strategies have been extensively applied in human infections but can be used in animal health. Antivirals are useful for an early control of virus spread after an outbreak. In addition, the combination of antivirals with vaccination protocols could be applied to elicit the immune response required for effective protection against the disease. Here, we have reviewed potential molecular targets to be considered as targets for antivirals. Antivirals against ASFV described include resveratrol and oxyresveratrol [73], microalgae [74], cholesterol lowering drugs [46] or inhibitors of cholesterol transport [42], antitumoral lauryl-gallate [75], anticonvulsivant valproic acid [75], dynamin inhibitors [38], fluoroquinolones [76], serine protease inhibitors [52], specific peptides [77], and miscelanea [32]. These could be eventually applied in a quick response to reduce susceptible animals in order to create ‘safe areas’ around the outbreaks and thus control the spread of the infection.
